# Phylogenetically Defined Isoforms of *Listeria monocytogenes* Invasion Factor InlB Differently Activate Intracellular Signaling Pathways and Interact with the Receptor gC1q-R

**DOI:** 10.3390/ijms20174138

**Published:** 2019-08-24

**Authors:** Yaroslava Chalenko, Egor Kalinin, Victor Marchenkov, Elena Sysolyatina, Alexey Surin, Konstantin Sobyanin, Svetlana Ermolaeva

**Affiliations:** 1 Laboratory of Ecology of Pathogenic Bacteria, Gamaleya Research Center of Epidemiology and Microbiology, Moscow 123098, Russia; 2 Laboratory of Molecular Microbiology, Nizhny Novgorod Research Veterinary Institute–Branch of Federal Research Center for Virology and Microbiology, Nizhny Novgorod 603022, Russia; 3 Laboratory of Protein Physics, Institute for Protein Research RAS, Puschino 142290, Russia; 4 Laboratory of Active Media, Moscow Institute of Physics and Technology, Dolgoprudnyi 141701, Russia; 5 Pushchino Branch, Shemyakin–Ovchinnikov Institute of Bioorganic Chemistry, Pushchino142290, Russia

**Keywords:** virulence, evolution, bacterial virulence factors, mammalian surface receptors, host-parasite interactions, c-Met, gC1q-R, Listeria, InlB

## Abstract

The pathogenic Gram-positive bacterium *Listeria monocytogenes* has been evolving into a few phylogenetic lineages. Phylogenetically defined substitutions were described in the *L. monocytogenes* virulence factor InlB, which mediates active invasion into mammalian cells via interactions with surface receptors c-Met and gC1q-R. InlB internalin domain (idInlB) is central to interactions with c-Met. Here we compared activity of purified recombinant idInlB isoforms characteristic for *L. monocytogenes* phylogenetic lineage I and II. Size exclusion chromatography and intrinsic fluorescence were used to characterize idInlBs. Western blotting was used to study activation of c-Met-dependent MAPK- and PI3K/Akt-pathways. Solid-phase microplate binding and competition assay was used to quantify interactions with gCq1-R. Isogenic recombinant *L. monocytogenes* strains were used to elucidate the input of idInlB isoforms in HEp-2 cell invasion. Physicochemical parameters of idInlB isoforms were similar but not identical. Kinetics of Erk1/2 and Akt phosphorylation in response to purified idInlBs was lineage specific. Lineage I but not lineage II idInlB specifically bound gC1q-R. Antibody against gC1q-R amino acids 221–249 inhibited invasion of *L. monocytogenes* carrying lineage I but not lineage II idInlB. Taken together, obtained results suggested that phylogenetically defined substitutions in idInlB provide functional distinctions and might be involved in phylogenetically determined differences in virulence potential.

## 1. Introduction

*Listeria monocytogenes* is a Gram-positive bacterium, which causes a severe foodborne disease, listeriosis [1]. Listeriosis manifests by sepsis, abortion, meningitis and meningoencephalitis. Listeriosis primarily affects elderly people and immunocompromised persons although the disease can also develop in healthy individuals [2,3,4]. Pregnant women represent one more group of risk, and perinatal listeriosis has severe consequences for fetus viability [5,6,7]. *L. monocytogenes* is a facultative intracellular pathogen. *L. monocytogenes* virulence is based on the ability to infect non-professional phagocytes such as epithelial and endothelial cells, hepatocytes, splenocytes, trophoblasts, etc. [8,9,10].

The species *L. monocytogenes* divides into four phylogenetic lineages, 13 serovars and multiple clonal complexes with different pathogenicity potential [11,12,13]. Lineage I strains are responsible for the majority of outbreaks and at least half of sporadic cases of listeriosis in humans and animals [14,15,16,17]. Lineage II strains are responsible for the majority of other listeriosis cases in humans and are overrepresented among animal and food isolates [14,18,19]. Strains of lineages III and IV are rare among human and animal isolates [20,21,22]. 

Most strains, regardless of which line they belong to, carry major virulence factors including Listeriolysin O, phospholipases, the transcriptional regulator PrfA encoded by the pathogenicity island LIPI-I and invasion factors of the internalin family InlA and InlB encoded by the *inlAB* operon [18,22,23,24,25]. These and a few other factors provide the intracellular phase in the *L. monocytogenes* life cycle [2,8,9]. The higher virulence potential of lineage I strains was suggested to be due to additional virulence factors that provide success in the course of infection of the macroorganism, such as Listeriolysin S, which is a bacteriocin from epidemic lineage I strains that targets the gut microbiota [26]. On the other hand, phylogenetically defined isoforms of major virulence factors might be another tool that is responsible for differences in the virulence potential of *L. monocytogenes* phylogenetic lineages. Point mutations inactivating the haemolysin Listeriolysin O (LLO) or the master-regulator PrfA were described that result in appearance of low virulent strains [25,27,28,29]. Premature stop codon in the *inlA* gene that result in appearance of truncated forms of the invasion factor InlA and decreased virulence are common among lineage II serovar 1/2a food isolates [28,29,30].

The virulence factor InlB mediates active invasion into many cell types [31]. Phylogenetically determined variations are characteristic for InlB as well for other virulence-associated proteins. Evolutionary analysis showed significant evidence for positive selection and recombination in the *inlB* gene [32]. Recently, we described distinct InlB isoforms characteristic for *L. monocytogenes* strains of the I and II phylogenetic lineages [33,34,35]. At least seven conservative lineage-specific amino acid substitutions were described in the internalin domain of InlB (idInlB, see below) [35]. Noticeable association with clonal complexes was observed for idInlB isoforms described in lineage I strains [33]. In lineage II strains, an idInlB isoform was revealed that is widely distributed among distinct clonal complexes. This isoform designated as idInlB14 was described in strains belonging to clonal complexes CC7, CC8, CC14, CC19, CC20, and some others [33,34]. Being placed in the same genetic background, InlB isoforms differently affected *L. monocytogenes* virulence in mice [35,36]. These data suggest that natural InlB isoforms could have some differences in their functionality.

InlB specifically interacts with the tyrosine kinase c-Met that results in activation of c-Met-controlled signaling pathways including MAPK-, PI3K/Akt- and STAT-controlled cascades [37,38,39]. Besides c-Met, InlB binds C1q-binding receptor gC1q-R and surface glycosaminoglycans [40,41]. InlB belongs to the so-called internalin family of proteins that are characterized by the internalin domain (id) composed of the LRR (for **l**eucine **r**ich **r**epeat) domain flanked by N-cap and immunoglobulin (Ig) -like domains [42,43]. The 321 amino acid InlB internalin domain (idInlB) is the minimal InlB part that interacts with and activates the tyrosine kinase c-Met [42,43,44,45]. The C-terminal GW-domains are required for InlB presentation on the bacterial surface and interact with gC1q-R and surface glycosaminoglycans [46,47].

gC1q-R known as well as p32 (or p33) is a multi-compartmental and multi-functional protein interacting with a wide range of ligands of endogenous and exogenous origin [48]. Surface presented gC1q-R has been reported to recognize and bind a number of functional antigens of viral and bacterial origin providing microbial attachment and/or entry [41,49,50,51]. Data on interactions of InlB with gC1q-R protein are controversial. On one side, a role of gC1q-R in InlB-mediated cell invasion is supported by competition experiments and experiments on adaptation of InlB-ignoring guinea pig cells to *L. monocytogenes* invasion by introduction of human gC1q-R [41]. On the other side, gC1q-R interactions with InlB C-terminal GW (for a conserved Gly-Trp (GW) dipeptide) domains stimulate release of InlB from bacterial surface and antagonize c-Met signaling induced by internalin domain/c-Met interactions [40,47].

Here we compared lineage I and lineage II idInlB variants. We demonstrated that while physicochemical properties of idInlB isoforms differed unessentially, they diverged noticeably in their potential to stimulate c-Met controlled signaling processes. Moreover, we demonstrated that idInlB found in strains of the clone complex CC1 of the I phylogenetic lineage interacted with gC1-qR while idInlB isoforms found in strains of the II phylogenetic lineage did not. Interactions between CC1 lineage I idInlB and gC1q-R were critical for *L. monocytogenes* invasion into human epithelial HEp-2 cells.

## 2. Results

### 2.1. Physicochemical Properties of Idinlb Isoforms

**Figure 1:**
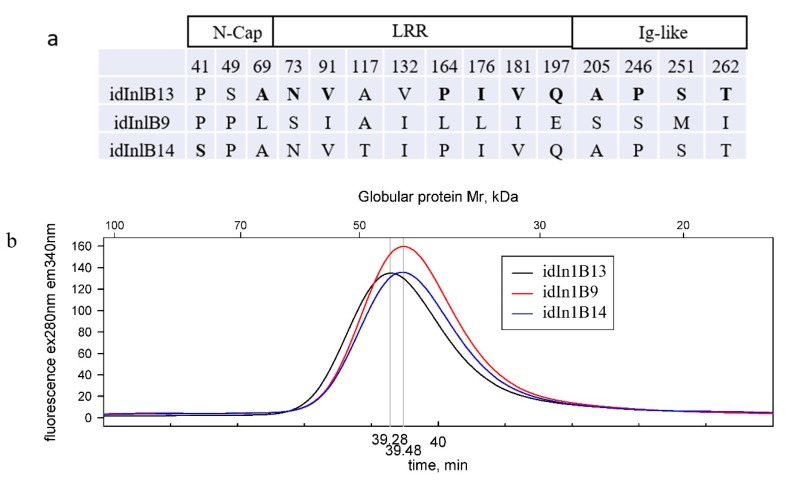

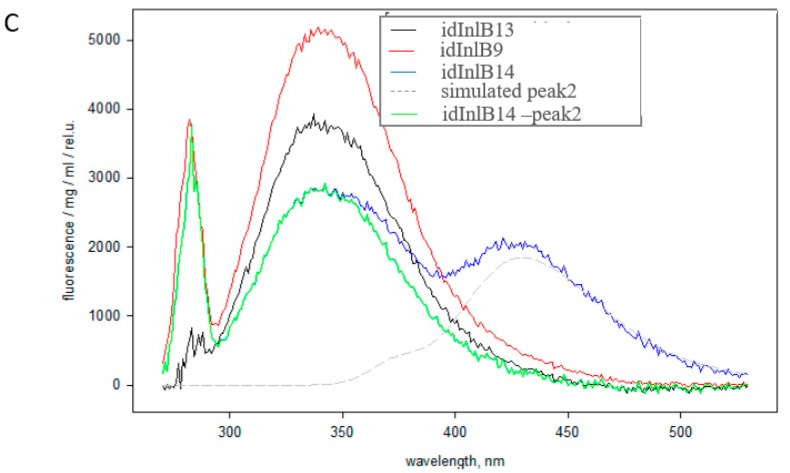
Physicochemical characteristics of idInlB isoforms: (**a**) Amino acid substitutions. The idInlB13 sequence was taken as a basis. Substitutions between lineages I and II idInlBs are shown in bold. LRR for **l**eucine **r**ich **r**epear; (**b**) size exclusion chromatography (SEC) of purified idInlBs; (**c**) fluorescence spectra of purified idInlBs. For idInlB14, original and retracted spectra are shown (see text for details).

A total of three InlB internalin domain (idInlB; amino acids 36–321) isoforms were included into the study (Figure 1a). One of them was specific for lineage I strains, and two others were specific for lineage II strains. The lineage I specific idInlB isoform designated as idInlB9 [35] was described in highly virulent strains of the epidemic clone ECI, clonal complex CC1, and cloned from the EC1 strain VIMHA015 (ST1, CC1) [52]. Among lineage II isoforms, the first designated as idInlB13 [35] was cloned from the type strain EGDe (CC9) [53] and the second designated as idInlB14 [35] was cloned from the human isolate VIMHA034 (CC7). Two lineage II forms were taken because the variant idInlB13 from the type strain EGDe is the best described and used in a number of studies [45,53] while idInlB14 is the most frequent among serovar 1/2a *L. monocytogenes* strains isolated from wild animals, clinical samples and other sources [33,34,54]. Moreover, a difference in their impact on virulence has been shown previously [35,36,55]. Amino acid substitutions which differentiate the variants are shown in the Figure 1a. idInlB13 and idInlB14 differed by four substitutions in positions 41, 49, 117 and 132. Eleven substitutions differed lineage I and lineage II variants (shown in bold at Figure 1a).

The purified proteins were compared by size exclusion chromatography (SEC) and intrinsic fluorescence. The SEC profiles of all variants included a major peak with the elution times corresponding to monomeric state of the proteins (Figure 1b). The higher quantum yield of fluorescence at 340 nm was observed for idInlB9 while peak intensities were comparable for idInlB13 and idInlB14. A slight shift in peak time position was observed for idInlB13 comparatively to other variants (elution times 39,28 < 39,48 = 39,48 min for idInlB13, idInlB9 and idInlB14, respectively).

To further assess protein conformation, intrinsic fluorescence spectra were taken. idInlB9 demonstrated the highest relative fluorescence. In contrast with the data above, fluorescence of idInlB14 and idInlB13 was different with idInlB14 demonstrating the lowest quantum yield. Moreover, the second peak was observed for idInlB14 with maximum at 440 nm. There is no amino acid with emission at this wavelength. Retraction of peak 2 showed that fluorescence of idInlB14 was very similar in shape to of other proteins although lower in intensity (Figure 1c).

### 2.2. Kinetics of Activation of MAPK and PI3K Signaling Pathways by idInlBs was Different foridInlB Isoforms

**Figure 2:**
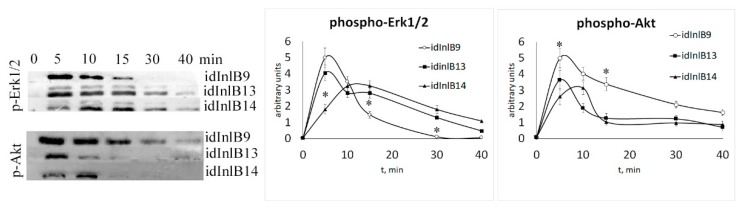
Kinetics of Erk1/2 and Akt phosphorylation in the presence of idInlBs. A total of 100 ng/mL idInlBs were added to HEp-2 cells. Cells were lysed at pointed time points and probed with anti-phospho-Erk1/2 and anti-phospho-Akt antibodies. The curves show digitized data from three independent experiments. * *p* < 0.05.

Next, we compared kinetics of phosphorylation of Erk1/2 and Akt kinases in HEp-2 epithelial cells treated with idInlBs. The Erk1/2 and Akt kinases represent the MAPK- and PI3K-controlled pathways, which are central signaling cascades triggered by c-Met ligand binding. We found noticeably different dynamics of phosphorylation of both Erk1/2 and Akt depending on what idInlB variant was applied (Figure 2). Both lineage II idInlB13 and idInlB14 caused a similar kinetics of phospho-Erk1/2 and phospho-Akt appearance at all time points but at 5 min. In contrast, addition of lineage I idInlB9 provided a different kinetics of Erk1/2 and Akt phosphorylation. Lineage II idInlBs caused phosphorylation of Erk1/2 kinase for more than 40 min while addition of lineage I idInlB9 caused Erk1/2 phosphorylation for not more than 30 min. In contrast, phosphorylation of Akt kinase was more prolonged upon addition of idIinlB9. Effect of lineage II idInlBs on Akt phosphorylation was less pronounced both in intensity and duration. During the first minutes after addition, idInlB14 caused noticeably different effectiveness of Erk1/2 and Akt phosphorylation compared to both other variants. For idInlB9 and idInlB13 maximal accumulation of phospho-Erk1/2 and phospho-Akt was observed 5 min post protein additions, while both phosphorylated kinases noticeably increased between 5 and 10 min post idInlB14 addition.

### 2.3. idInlBs Isoforms Interacted with gCq1-R In Vitro in a Lineage-Specific Manner

**Figure 3:**
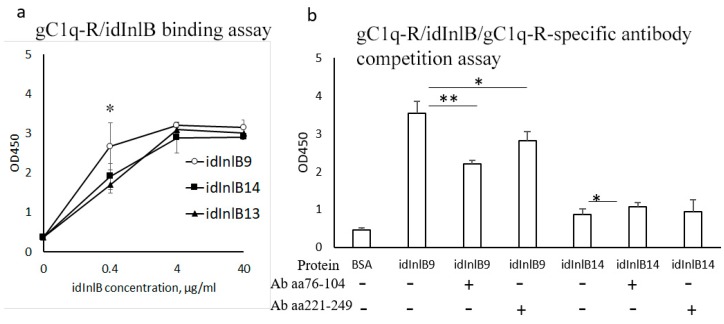
In vitro study of interactions between idInlBs and gC1q-R using solid-phase microplate binding and competition assay. Microtiter plate wells were coated with recombinant human gC1q-R. Purified idInlBs were added. ELISA assay was performed with His-tag-specific polyclonal antibodies and HRP-conjugated secondary antibodies. (**a**) Saturation curve demonstrating binding of three idInlB isoforms to gC1q-R; purified idInlBs were added in concentrations 0–40 µg/mL: circles-idInlB9, triangles-idInlB13, squares-idInlB14; (**b**) a competition assay; polyclonal antibodies developed against oligopeptides specific for central (amino acids 76–104) and C-terminal (amino acids 221–249) parts of gC1q-R were added at concentration of 4 µg/mL 1 h before idInlBs were added in concentration 0.4 µg/mL. BSA was used as a negative control, and idInlBs without antibodies were used as positive controls. Mean ± SD from three experiments made in triplicate are shown; * *p* < 0.05; ** *p* < 0.01. Statistical significance of competition experiments relative to a corresponding positive control is shown.

To address whether idInlBs interacted directly with gC1q-R, we developed an ELISA assay to compare interactions between idInlB isoforms and gC1q-R. All idInlB variants interacted with gC1q-R in vitro although the specificity of interactions appeared to be different. When taken in the concentration of 0.4 µg/mL, lineage I specific idInlB9 bound gC1q-R proteins better than both lineage II idInlBs (Figure 3a). At concentrations higher than 0.4 µg/mL effectiveness of gC1q-R binding was similar for all isoforms.

To check whether interactions between idInlBs and gC1q-R were specific, a competitions assay was performed. idInlB14 (lineage II) was used in this assay because both lineage II idInlBs behaved similarly in the binding assay. idInlB14 was compared with lineage I specific idInlB9. Both idInlB were taken in concentration of 0.4 μg/mL. Antibodies protected the central region of human gC1q-R (amino acids 76–104) and protected the C-terminal region of gC1q-R (amino acids 221–249) were used to block idInlB binding. Binding of idInlB9 was partly blocked by the antibody developed against the central region of human gC1q-R (by a factor of 1.6, *p* < 0.01; Figure 3b) and to a lesser extent by the antibody against the C-terminal region of gC1q-R (by a factor 1.25, *p* < 0.05). Binding of idInlB14 was independent of antibodies.

### 2.4. gC1q-R Antibodies Specifically Inhibited Lineage I idInlB- but not Lineage II idInlB-Driven Invasion into HEp-2 Cells

**Figure 4:**
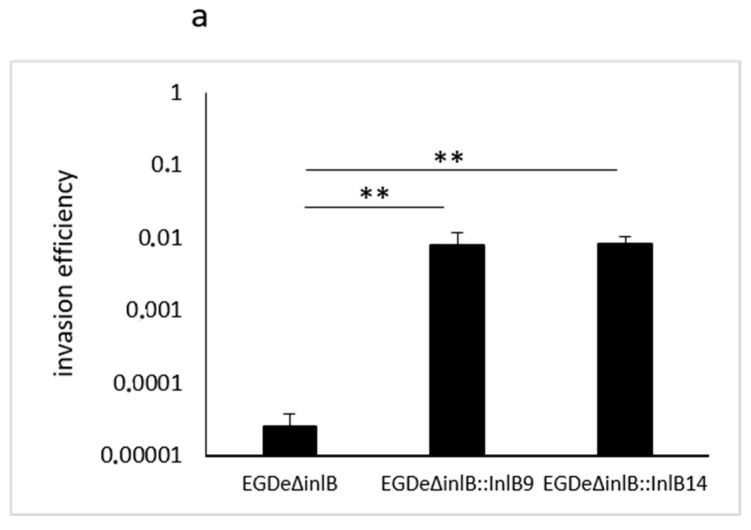

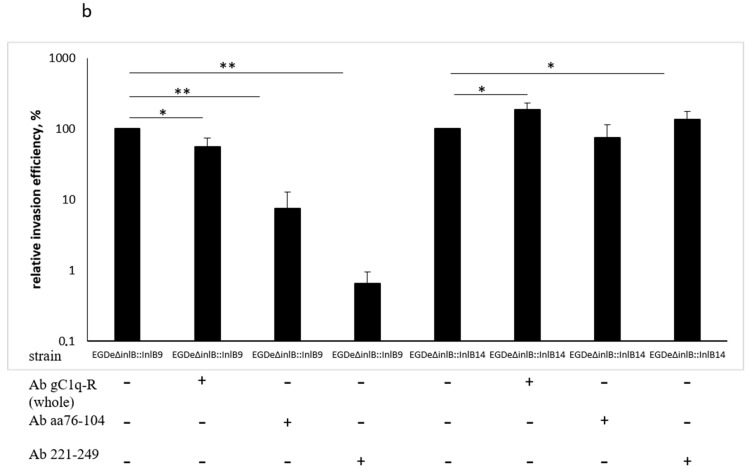
Invasion efficiency of idInlB9- but not idInlB14-carrying *L. monocytogenes* was gC1q-R dependent. Gentamicin invasion assay was performed as described in the materials and methods sections. Isogenic strains EGDeΔinlB (parental strain lacking the *inlB* gene); EGDeΔinlB::InlB9 and EGDeΔinlB::InlB14 were used to infect human HEp-2 epithelial cells with MOI 100:1 (bacteria:cells). (**a**) Invasion efficiency of isogenic strains; invasion efficiency was calculated as a ratio of intracellular bacteria to bacteria used for infection; (**b**) inhibition assay; polyclonal antibody against the whole gC1q-R protein or polyclonal antibodies developed against oligopeptides specific for central (amino acids 76–104) and C-terminal (amino acids 221–249) parts of gC1q-R were added in concentration of 4 µg/mL 1 h before isogenic recombinant *L. monocytogenes* strains EGDeΔinlB::InlB9 and EGDeΔinlB::InlB14 were added; relative invasion efficiency is shown that was calculated as a percentage of invasion efficiency relative to a positive control (invasion efficiency of a strain EGDeΔinlB::InlB9 or EGDeΔinlB::InlB14 without any antibodies). Results represent mean ± SD from three experiments made in duplicate; * *p* < 0.05; ** *p* < 0.01. Statistical significance of competition experiments relative to a correspondent positive control is shown.

To check how specific binding to gCq1-R could affect InlB-driven cell invasion, we performed “gentamicin invasion assay” using human epithelial HEp-2 cells and isogenic recombinant *L. monocytogenes* strains. The strains were constructed on the basis of the strain EGDeΔinlB lacking the *inlB* gene (kindly provided by Prof. J. Vazquez-Boland) and expressed full size InlB variants that differed in idInlB only. Other InlB parts including the signal peptide, B-repeat and GW-domains as well as the promotor region controlling *inlB* gene expression were the same and taken from the strain EGDe [35]. Both full size InlBs, InlB9 (carrying idInlB9) and InlB14 (carrying idInlB14) improved invasion of the parental strain EGDeΔinlB (Figure 4a). To evaluate an impact of interactions between InlB variants and gC1q-R on bacterial invasion, HEp-2 cells were pre-treated with gC1q-R specific antibodies. Besides antibodies tested in vitro, the polyclonal antibody developed against the whole gC1q-R protein was used. For the *L. monocytogenes* strain EGDeΔinlB::inlB9, pre-treatment with antibodies against whole gC1q-R, its central part (amino acids 76–104) and its C-terminal part (amino acids 221–249) reduces invasion efficiency 2-, 13- and 153- fold, respectively (*p* << 0.05, Figure 4b). The last antibody specific to the C-terminal amino acids 221–249 totally inhibited invasion. It diminished invasion efficiency almost to the level of the strain EGDeΔinlB (0.000051 ± 0.000023 vs. 0.000025 ± 0.000013 for strain EGDeΔinlB::inlB9 in the presence of the antibody and strain EGDeΔinlB, respectively, *p* = 0.27). Antibodies provided different effects on invasion of the strain EGDeΔinlB::inlB14. Antibodies against the gC1q-R central part (amino acids 76–104) did not affect invasion (*p* = 0.329). In contrast, antibodies against the whole protein and the C-terminal part (amino acids 221–249) improved invasion in 1.9 and 1.3-times (*p* < 0.05).

## 3. Discussion

Here we demonstrated that phylogenetically defined isoforms of the *L. monocytogenes* invasion factor InlB internalin domain (idInlB) noticeably differed in their functional properties. Three idInlB isoforms were compared. The idInlB9 variant is characteristic for highly virulent CC1 strains of the phylogenetic lineage I [33]. In general, lineage I-specific idInlB isoforms demonstrated a tight association with clonal complexes. The idinlB9, idinlB1 and idinlB8 isoforms were shown to be characteristic for clonal complexes CC1, CC2 and CC3, respectively [33,35]. Five and one amino acid substitutions differ idInlB9 and idInlB1, and idinlB9 and idinlB8, respectively [35]. All three listed clonal complexes are strongly associated with strains of clinical origin [18]. The idilnB9 isoform was chosen because (i) the epidemiological significance of CC1 is well-known, and CC1 strains caused outbreaks in many countries including Russia; (ii) CC1 but not CC2 or CC3 strains are predominant in cases of neurological infection in ruminants suggesting that CC1-specific virulence features provide the high virulence for different hosts; (iii) idInlB9 is most divergent from lineage II idInlB isoforms [16,18,33,35]. In total, 11 amino acid substitutions were distinct for idinlB9 and lineage II idInlB isoforms used in this study. Previous studies showed that seven of these 11 substitutions are conservative distinctions between lineage I and lineage II strains [35]. Lineage II idInlB13 is characteristic for the type strain EGDe and CC9 strains that are strongly associated with a food origin and minor among clinical isolates, idInlB14 is found in a wide range of lineage II strains belonging to the serovar 1/2a and different clonal complexes (CC7, CC8, CC14, CC19,CC 20, CC21, CC155, CC177 and some others) [18,33,34]. Four amino acids differentiated idInlB13 and idlnB14 isoforms.

Polymorphism of virulence factors is well-established in pathogenic bacteria. In previous years, experimental evidence has accumulated on functional differences that provide a role for phylogenetically defined isoforms of virulence factors in development of highly virulent clones of different bacterial species. Isoforms of the *Salmonella enterica* SpvD protease, which negatively regulates the NF-κB signaling pathway, differ by substitutions in the position 161 [56,57]. The SpvD^Gly161^(SpvD with Gly at the position 161) variant characteristic for the Enteritidis serovar was shown to more potently inhibit NF-κB-mediated immune responses in cells in vitro than the SpvD^Arg161^ (SpvD with Arg at the position 161) variant characteristic for the serovar Typhimurium. Introduction of the SpvD^Gly161^ variant in the Typhimurium strain increased its virulence in mice [57]. *Staphylococcus aureus* Panton-Valentine leukocidin (PVL) toxin has two isoforms, R and H with different geographic distribution [58,59]. Most methicillin-resistant *S. aureus* strains distributed in USA belong to the R groups. The molecular model was suggested that H-R substitution might have functional implications that result in better fitness for the R variant, thus contributing to the successful spread and high virulence of the USA300 strain [58]. The epidemic highly virulent *Yersinia pestis* strains possessed only the Pla^T259^ isoform of the plasminogen activator Pla while Pla^I259^ isoform is spread among *Y. pestis* isolates with limited virulence [60,61]. Computational and experimental analysis demonstrated that the Pla isoform found in *Y*. *pestis* epidemic strains is more functionally efficient than that of the low virulent strains [61,62],

In line with these results, our data demonstrated that phylogenetically defined substitutions in the *L. monocytogenes* invasion factor InlB were implemented in functional differences between idInlB isoforms. Lineage I and lineage II specific idInlB isoforms differentially activated c-Met-dependent MAPK- and PI3K/Akt- signaling pathways and interacted with gC1q-R protein.

The most evident difference between idInlB isoforms was a different potential for interactions with the gC1q-R receptor, the multifunctional mammalian protein presenting in different compartments including the cell surface. In a solid-phase microplate binding assay, gC1q-R bound CC1-specific idInlB9 better than lineage II specific idInlB13 and idInlB14. However, when ligands were taken in excess gC1q-R bound both lineage I and lineage II idInlBs. gC1q-R is a highly acidic, rather “sticky” protein that interacts with multiple ligands of cellular, protozoan, bacterial and viral origin [41,48,49,51,63]. Trying to exclude unspecific interactions between gC1q-R and idInlBs we applied an in vitro competition assay. Antibodies specific to the central and C-terminal parts partly inhibited interactions of gC1q-R with idInlB9 but not with idInlB14.

The in vivo invasion assay was in line with in vitro results. Isogenic *L. monocytogenes* strains expressing recombinant full length InlB were used. Recombinant InlBs differed in idInlB only while they carried the same signal peptide and GW-domains. The competition invasion assay demonstrated that a polyclonal antibody to amino acids 221–249 at the C-terminal part of the human gC1q-R totally repressed idInlB9-driven invasion into human epithelial HEp-2 cells decreasing it 150-fold, but did not affect invasion driven by lineage II specific idInlB14. Invasion in the presence of another antibody specific to amino acids 76–104 at the central gC1q-R part was partly inhibited for lineage I but not lineage II carrying *L. monocytogenes*. Interestingly, that polyclonal antibodies developed against the whole gC1q-R protein decreased invasion efficiency of idInlB9-carrying strain only two-fold suggesting site-specific interactions of gC1q-R with idInlB9. The important for interactions region 221–249 is located in the larger gC1qR segment encoded by residues 159–282 which also contains the site for HCV core protein [63]. In contrast to idInlB9, idInlB14-driven *L. monocytogenes* invasion that was not inhibited specifically by antibodies against gC1q-R. Statistically significant 1.9-fold increase in efficiency of idInlB14-driven invasion was provided by application of the polyclonal antibodies to gC1q-R. This effect might be prescribed to inhibition of interactions between gC1q-R and GW-domains of the full length InlB as it was shown previously [47]. These interactions were suggested to affect GW-domain dependent InlB binding to the bacterial surface that negatively affects *L. monocytogenes* invasion [47].

Obtained results suggested that interactions of idInlB with gC1q-R are important for virulence of CC1 strains but seem to be of less importance for lineage II *L. monocytogenes* strains. The importance of interactions with gC1q-R for lineage I strains belonging to other clonal complexes should be checked. gC1q-R is a multifaceted protein that is particularly involved in control of cytoskeleton rearrangements during lamellipodia and filopodia formation [64]. This function might be important for the role of interactions between gC1q-R and idInlB in *L. monocytogenes* active cell invasion. Results obtained in this work are in line with our previous results demonstrating differences in lamellipodia and filopodia formation induced by different idInlB isoforms in HEp-2 cells [65]. The known role of InlB as not only an invasion factor but also as a endocytosis activating factor [66] provides a direction for further studies on functional roles of its phylogenetically defined variability.

Overall, obtained results demonstrated differences in functional activities of phylogenetically defined idInlB isoforms. These differences are in line with previously obtained data that demonstrated that isogenic recombinant *L. monocytogenes* strains expressing full length InlB variants differed by idInlB only have different virulence for mice [36]. Taken together, these observations suggested that idInlB isoforms might provide different effects on *L. monocytogenes* virulence, that in turn might be one of foundations of the different virulence potential of *L. monocytogenes* phylogenetic lineages and/or distinct clonal groups.

## 4. Materials and Methods

### 4.1. Bacterial Strains and Growth Conditions

*Escherichia coli* expressing strains BL21::pET28b(+)::InlBallele9, BL21::pET28b(+)::InlBallele13 and pET28b(+)::InlBallele14 were used [52]. Shortly, the strains were obtained by cloning the idInlB (amino acids 36–321) encoding *inlB* gene fragment from the *L. monocytogenes* strains VIMHA015 (serovar 4b, clinical isolate) for BL21::pET28b(+)::InlBallele9, EGDe (serovar 1/2a, type strain) for BL21::pET28b(+)::InlBallele13, and VIMHA034 (serovar 1/2a, clinical isolate) for BL21::pET28b(+)::InlBallele14 into the pET28b(+) vector (Novagen^®^, a brand of EMD Biosciences, Inc., an affiliate of Merck KGaA, Darmstadt, Germany). The idInlB proteins are referred as idInlB9 (for the protein produced by the BL21::pET28b(+)::InlBallele9 strain), idInlB13 (for BL21::pET28b(+)::InlBallele13), and idInlB14 (for BL21::pET28b(+)::InlBallele14). Allele numbering was given in accordance with [33,34].

Construction of isogenic recombinant *L. monocytogenes* strains on the basis of the strain EGDeΔinlB lacking the *inlB* gene (the strains were generously provided by Prof. J. A. Vazquez-Boland, Univ. Edinburgh) was described previously [35]. Shortly, idInlB (amino acids 36–321) encoding *inlB* gene fragments like those that were used for construction of *E. coli* expressing strains, were incorporated into the vector pInlAB made on the basis of the shuttle vector pTRKH2 [67] by introduction of the promoter of *inlAB* operon, an *inlA* gene fragment ending a signal peptide required for protein secretion, and *inlB* gene fragment encoding the B-repeat and GW-domains all fragments from the *L. monocytogenes* strain EGDe, Lineage II [53]. Introduction of the idInlB encoding gene fragment restored the full length *inlB* gene. The strains carrying idInlB9 and idInlB14 were designated EGDeΔinlB::inlB9 and EGDeΔinlB::inlB14, respectively [35]. The recombinant proteins InlB9 and InlB14 carried lineage-specific idInlB domains while they shared the same N-terminal signal peptide and the C-terminal B-repeat and GW-domains.

Plasmid-carrying *E. coli* strains were grown on the LB agar or broth medium (Sigma-Aldridge, St. Louis, MS, USA), supplemented with Km (100 µ mL^−1^). Recombinant *L. monocytogenes* was grown on the BHI agar or broth medium (BD, Franklin Lakes, NJ, USA) supplemented with Em (10 µ mL^−1^). 

### 4.2. Protein Purification

The proteins with the total length of 330 aa were purified via a His-tag with Dynabeads (Invitrogen^®^, the brand of Thermo Fisher Scientific, Waltham, MA, USA) ), subjected to dialysis with Slide-A-Lyzer™ MINI unit (Thermo Fisher Scientific,) against the Na-phosphate buffer (50 mM Na-phosphate buffer, pH 8.0, 300 mM NaCl), and stored at +4 °C at the concentration of 1.5 mg/mL.

### 4.3. Cell Culture and Growth Conditions

Human epithelial HEp-2 cells obtained from the collection of Gamaleya Research Center were grown in a DMEM medium (PANEKO, Moscow, Russia) with 10% fetal bovine serum (Gibco^®^, the brand of Thermo Fisher Scientific, Waltham, MA, USA) in a 5% CO_2_ atmosphere at 37 °C.

### 4.4. Size Exclusion Chromatography (SEC)

SEC was carried out in a PBS buffer on a Superdex 200 10/300 GL (GE Healthcare Bio-Sciences AB, Uppsala, Sweden) column attached to a ProStar HPLC chromatograph (Varian, Palo Alto, CA, USA). Elution was performed at a flow rate of 0.4 mL/min. The elution was monitored by fluorescence emission at 340 nm when excitation light wavelength was set to 280 nm.

### 4.5. Fluorescence Spectra

Fluorescence spectra were measured in a square quartz cuvette 3 × 3 mm with a Cary Eclipse spectrofluorometer (Varian, Palo Alto, CA, USA) at protein concentration in a PBS buffer 0.03 mg/mL. Excitation wavelength was 280 nm, and both excitation and emission slits were set to a bandwidth 10 nm.

### 4.6. Immunoblotting

idInlB9 or idInlB14 was added to HEp-2 cells grown as described above up to a concentration of 100 ng/mL. At time points described in the text, cells were resuspended in 100 µL RIPA buffer (Thermo Fisher Scientific) supplemented with protease and phosphatase inhibitor cocktails (Sigma Aldrich). Cell lysates were boiled for 10 min, separated on 10% SDS-PAGE and transferred onto PVDF membrane (Amersham, the brand of GE Healthcare, Chicago, IL USA)). Phospho-Erk1/2 and phospho-Akt were visualized with primary antibodies PA5-37828 and 44-621G, respectively (Thermo Fisher Scientific) and secondary HRP-labelled ab97085 antibodies (Abcam PLC, Cambridge CB2 0AX UK). The optical density of each band was measured using TotalLab 1.10 software and presented graphically in arbitrary units. 

### 4.7. Solid-Phase Microplate Binding and Competition Assay

The ability of various idInlB bind gC1q-R protein was assessed by solid-phase ELISA using microtiter plates (Thermo Fisher Scientific, #15041). The assay was performed according to manufacturer instructions. Briefly, after pre-treatment with 2% glutaraldehyde in PBS, pH 5.0, and washing with TTBS (20 mM TrisHCl, pH 7.5, 150 mM NaCl, 0.05% Tween-20), triplicate wells were coated (overnight at +4 °C) with 100 μL C1QBP Recombinant Human Protein (Invitrogen, #11874H08E25), 4 µg/mL in 0.1 M sodium carbonate buffer, pH 9.4. The unbound proteins were aspirated, the wells were washed with TTBS and the unreacted sites were blocked by SuperBlock^TM^ Blocking Buffer (Thermo Fisher Scientific, #37515) supplemented with 0.05% Tween-20 at room temperature for 1 h. To block C1QBP His-Tags, plates were incubated with 6x-His Tag Monoclonal Antibody (4E3D10H2/E3; Thermo Fisher Scientific **#** MA1-135) for 1 h. When competition assay was performed, gC1q-R specific polyclonal antibodies were added at the same stage. Two antibodies were used: #PA5-14989 specific to amino acids 76–104 from the central region of human gC1q-R, and # PA5-14988 specific to amino acids 221–249 from the C-terminal region of human GC1qR (both antibodies from Thermo Fisher Scientific). The unbound antibodies were washed away, and purified idInlB proteins diluted in a Blocking Buffer in concentrations (0–40 µg/mL) were added and plates were incubated at room temperature for 1 h. Bound idInlB was revealed with 6x-His Tag Polyclonal Antibody (Thermo Fisher Scientific #PA1-983B) that interacted with idInlB His-Tags and HRP-conjugated secondary antibodies Anti-Rabbit IgG H&L (HRP) (Abcam pre-adsorbed; # ab97085). HRP activity was stained with 1-Step Ultra TMB-ELISA substrate (Thermo Fisher Scientific, #34028). The absorption was measured at λ = 450 nm. The washing stages between the reactions were performed three times with TTBS.

### 4.8. Invasion Assay

Invasion assay was performed as described in [35]. Briefly, 70% HEp-2 cell monolay was infected with mid-exponential bacteria (MOI 100:1, bacteria:cells). After 1 h incubation, the monolay was washed with PBS and gentamicin (Fluka, the brand of Thermo Fisher Scientific) was added up to a concentration of 100 µg/mL. One-hour later, cells were washed with PBS and lysed with 1% Triton X-100 (Sigma). Decimal dilutions of cell lysates were plated on BHI. Colonies were counted 24 h later. In some experiments, gC1q-R specific antibodies (#PA5-14989; #PA5-14988; or #PA5-55318 developed against recombinant protein corresponding to human gC1q-R; all antibodies from Thermo Fisher Scientific) were added up to concentration 4 µg/mL 1 h before bacterial infection. Invasion efficiency was calculated as a ratio of intracellular bacteria to the bacteria applied to cells. Relative invasion efficiency was calculated as a percentage of invasion efficiency relative to invasion of bacteria in absence of antibodies.

### 4.9. Statistics

All experiments were performed using duplicate or triplicate samples and repeated at least three times. To evaluate the data obtained in vitro, the mean values and SD were calculated with Excel software (Microsoft Office 2010). The paired t-test included in the same software was used for assessment of statistical significance. Two-group comparisons were conducted using the Student’s t-test. A value of *p* < 0.05 was considered to indicate a statistically significant difference.

## Abbreviations

CC	Clonal complex
id	Internalin domain
idInlB	InlB internalin domain
SEC	Size-exclusive chromatography
ST	Sequence type
